# Supplementary material for: Exploring the complex nature of implementation of Artificial intelligence in clinical practice: an interview study with healthcare professionals, researchers and Policy and Governance Experts
Source: PLOS Digit Health. 2025 May 7;4(5):e0000847. doi: 10.1371/journal.pdig.0000847 (PMC12057897; doi:10.1371/journal.pdig.0000847)
Supplement: S1 Appendix — (DOCX) [file pdig.0000847.s001.docx]

**SUPPORTING INFORMATION**

**S1 Appendix. Interview guide**

**Introduction**

- Brief introduction of the researchers
- Explaining objective of the study and aim of the interview
- Explanation of ethical aspects and follow-up steps after the interview.
- Explanation recording of the interview
  - Transcripts
  - Anonymous
- Explanation structure of the interview
- Explain the Innovation Funnel

- Can you tell me something about your experience with AI in healthcare (using the use case)?
- If you were to rate your experience at the Funnel stage; can you indicate to which stage your experience extends?
- What prompted the development of the solution?
- How did the current work process run?
- What are the relevant stakeholders?
  - How did you identify them?
  - How were they included in the development and pilot phase?
- What was the intended end goal and impact of the solution? Did this change after development?
- How did you think about scaling up this solution?
  - Can you tell something about the business case?
  - In what way have you looked at the current market for the purpose of cooperation or competition?
- Who are the intended end-users? What does this end situation look like? Are these profiles also documented?
  - How do you include them in the development process?
  - How did you take into account the end user's capability (skills, role and task and context)?
  - What impact has this had on training and education?
- What design requirements have you included in terms of user experience and interaction?
- How did you understand how end users interact with the solution in the workflow? (patient journey, prototype)
  - To what extent did you consider the margin of error in the outcomes and how they are captured in practice?
- What data served as the source for your solution and what trade-offs did you make in it?  In terms of:
  - Availability
  - Reliability
  - Ownership
- Did you purchase or develop the technology and what were your trade-offs therein?
- What is documented about the data (collection, storage, processing) and the algorithm?
- Has consideration been given to how the final solution fits into the existing architecture?
- Think about governance and laws and regulations. To what extent have you taken these into account?
- Is there an organisation-wide way of shaping this kind of project?
- How does your project relate to governance in this area?
- To what extent is your project known within your organisation?
- Which relevant experts have you reached out to and about? And what were your experiences with them? Examples:
  - Privacy
  - Information security
  - Ethics
  - Legal issues
  - Medical legislation
- Can you tell me about risk assessments and liability
  - To what extent does your solution fall under the legislation of the MDR and what are the implications?
  - How is the clinical evaluation plan and report aligned with this?
  - Who is the manufacturer of the solution and how was this determined?

With the previous discussion: what do you think is the biggest challenge to developing valuable AI for healthcare? 
