# Supplementary material for: Exploring the complex nature of implementation of Artificial intelligence in clinical practice: an interview study with healthcare professionals, researchers and Policy and Governance Experts
Source: PLOS Digit Health. 2025 May 7;4(5):e0000847. doi: 10.1371/journal.pdig.0000847 (PMC12057897; doi:10.1371/journal.pdig.0000847)
Supplement: Attachment — Submitted filename: 20250116 Rebutall .docx [file pdig.0000847.s003.docx]

**Response to Reviewer Comments to the Author**

We thank the reviewers' thoughtful and constructive feedback on our manuscript. We appreciate the time and effort that the reviewers have invested in assessing our work. We have carefully considered the points raised and have made the following revisions to address the reviewers' comments point-by-point. We believe these revisions have significantly strengthened our manuscript.

**Reviewer #1**

Dear authors,
Thank you for your work on an interesting and highly relevant topic. In my opinion, there are some points that can be improved. These are detailed below.

1. Line 101: Please indicate the region in which this applies.

We have revised the manuscript to specify that the region in which the diagnostic applications of medical imaging are most prevalent.

Revised: Page 5, Line 103-105

*One of the leading fields is diagnostics by medical imaging, for instance, in detection and prediction of cancer, COVID-19 identification, diabetes and cardiology in countries across Asia and Europe.(5-10)*

2. Line 148: I'm wondering whether policy-makers is the right term for this group.

We agree that "policy makers" may not be the most appropriate term. Therefore, we have chosen "Policy and Governance Experts" as a more suitable term for the group we describe. This term encompasses a broad category of individuals involved in developing and implementing policies, including board members, managers, policy advisors, and legal experts. This group plays a crucial role in shaping strategic decisions and policy guidelines within our context but is broader than "decision makers.

Revision: We have changed this throughout the manuscript and in the title.

3. Line 179: You should explicitly mention the qualitative data analysis approach you have chosen. Please justify the approach used.

We chose thematic analysis because it provided the openness, flexibility, depth, and rigor needed to explore the complex and multifaceted nature of AI implementation in clinical practice. We used the IFVAIH funnel in the data collection, but this analysis method enabled us to adapt our analysis to the themes that emerged during the coding process and so to explore unexpected insights and patterns that might not have been captured if we had strictly adhered to the IFVAIH funnel. We have clarified this in the manuscript:

We chose thematic analysis because it provided the openness, flexibility, depth, and rigor needed to explore the complex and multifaceted nature of AI implementation in clinical practice. While we used the IFVAIH funnel in data collection, this analysis method allowed us to adapt our approach to the themes that emerged during the coding process. This flexibility enabled us to uncover unexpected insights and patterns that might not have been captured if we had strictly adhered to the IFVAIH funnel.

Revised: Page 8, Line 192-193

We used this approach to maintain openness and flexibility in identifying emerging themes based on the codes during the analysis of the data collected.

Page 23, Line 586-590

*Another limitation is that this study used IFVAIH as the framework for the interview guide to understand the implementation of AI in clinical practice.(37) This model is specifically designed to provide practical guidance for AI implementation and while we used the IFVAIH funnel in data collection, the thematic analysis method allowed openness and adapt our approach to the themes that emerged during the coding process. This flexibility enabled us to uncover unexpected insights and patterns that might not have been captured if we had strictly adhered to the IFVAIH funnel.*

4. Line 192: Make sure that the categories mentioned here are identical to the headings later on. In addition, it would be great to get an overview of all categories (“themes”) including subcategories (if there are any).

We have ensured that the categories mentioned in Line 192 are now identical to the headings used later in the manuscript. Additionally, we have provided an overview of all categories along with any subcategories in one table (Table 2) to enhance clarity and coherence for the readers. This should help readers better understand the structure and organization of our analysis.

Revised: Page 10, Line 214-219

*From these interviews we extracted six key themes: 1) Balancing between demand-pull and tech-push, 2) Focus on generating knowledge, not implementing working solutions, 3)Lacking multidisciplinary collaboration, 4) Lack of appropriate knowledge and skills, 5) The role of the hospital in AI-development and 6) Opportunities and challenges from evolving laws and regulations.*

5. Line 201: You should briefly describe, what is meant by “Balancing between demand-pull and tech-push”. I assume that both of these terms were developed by you and were not directly mentioned by any of the interviewees.

The theme 'balancing between demand-pull and tech-push' emerged from our thematic analysis (described in data-analysis of the Methods section (Page 7-9, Line 172-210) as a synthesis of interviewees' discussions about the balance between meeting user-driven needs (demand-pull) and advancing technology-driven solutions (tech-push). Although these specific terms were not directly mentioned by the interviewees, they were developed during the analysis to encapsulate the recurring ideas present in the data. To ensure the validity of this interpretation, we cross-checked the theme with other members of the research team familiar with the dataset and member checked this with the participants. See also comment below for revisions.

6. Line 226: Is this quote really appropriate for the “tech-push” here? Please also check the quote in lines 239 and 245. It seems to me that you have a very broad understanding of demand, which should be specified.

We have clarified our understanding of demand-pull and tech-push based up on the interviews in the first section of this theme. Additionally, we adapted our quotes to improve understanding for the readers.

Page 10, Line 226-234

*‘The push to experiment with the AI technology came from a technological perspective: can this specific task be done using AI? By 'demand-pull,' we refer to innovation driven by user needs, while 'tech-push' refers to innovation driven by technological advancements without explicit demand from end-users. The following quote illustrates the 'tech-push' approach, where the drive to use AI originated from ongoing technological efforts rather than a clinical demand: 'We were already working on AI and then we thought that it might also have potential for better diagnosis in the other patient group. (…) However, the potential room for improvement of our current method for this other patient group seem to be minimal ' (Participant #2, healthcare professional).’*

Page 11, Line 251-257

*Furthermore, researchers and healthcare professionals mentioned the tech-push may lead to the risk of non-valuable care delivery by the implementation of too inaccurate AI models. One participant said: ‘This tech-driven approach can be problematic. A lot of physiological processes things can be normal and not necessarily a sign of disease, right? For example, polyps: 90% of polyps are benign and certain benign polyps need treatment while the majority others don't. So, yes, if the AI tool for detection of maligne polyps is not accurate enough, it will lead to overdiagnosis rather than underdiagnosis and eventually overtreatment.’ (Participant #3, healthcare professional)*

Page 12, Line 263-273

*In addition, a demand-pull factor for healthcare professionals to get involved in AI development could be when the declining quality of care and longer waiting lists due to staff shortages and increase of patient volumes could add to the urgency of incorporating AI to maintain the accessibility of care for their patients. One participant said about demand-pull for patients and professionals: ‘Staff shortage plays a big role here. What would you do when there is no staff to help you with your complaint? Receiving no diagnosis at all or an AI diagnosis?’ I think then majority of patients and professionals would be preferring an AI diagnosis. (Participant #9, Policy and Governance Expert). Another healthcare professional said the following about demand-pull from professional perspective: ‘Take image recognition as an example. I have had to wait until 12 a.m. for the radiologist's results. If we could rely on an AI assessment, I could have gone home much sooner and I experience less workload.’ (Participant #3, healthcare professional)*

7. Line 275: Please specify what "your project" means. Between the three sub-groups I would think it means quite different things. Overall, it would be interesting if similarities and differences between the subgroups were mentioned for all results.

Revised: We have replaced 'project' with 'initiative' throughout our manuscript, as it more accurately represents what we mean. Additionally, we have clarified the definition of the initiatives in the methods section. Furthermore, we added the type of participant per quote in the results sections and if similarities and differences between the subgroups were mentioned for all results.

Page 7: Line 153-162

*Setting and participants*

*The study was conducted in a 942-bed tertiary teaching hospital in the Netherlands with ample experience in AI development. Participants with experience with AI development, research and/or implementation in their practice were eligible to be interviewed. We defined three subgroups of participants: healthcare professionals (physicians, nurses), researchers (medical technology, technical medicine, data scientists, PhD students) and Policy and Governance Experts (members of the board, managers, policy advisors, legal experts). Inclusion criteria were having experience with at least one AI initiative as a use case of AI development in their current job position/specialism, and willingness to participate in the study. To explore a broad range of experiences with AI, we interviewed a purposefully sampled group of employees per subgroup.*

8. Line 396: I would recommend that you summarize the main findings (sub-categories) before going into detail.

Revised: We have added an overview table (Table 2, Page 13) where we summarize the themes and subcategories to promote the clarity for the readers. In addition, we mapped the subthemes onto the domains of the IFVAIH funnel to enhance understandability of the themes in regard to discussion section (key finding, implications sections).

9. Line 424: In my opinion, it is not necessary for you to repeat the findings. It would be more interesting if you synthesized all the themes to find common patterns or relevant contextual factors. On this basis, you could improve the next section by not only comparing your findings with those of other studies, but also by providing explanations and theoretical assumptions.

Revised: The synthesis of the data is an integral part of the thematic analysis described in the Results section. In the Discussion section, we have rewritten the key findings to provide a more high-level overview, focusing on the patterns in relation to the IFVAIH funnel rather than listing them.

Additionally, we have expanded the comparison with prior work section, particularly in relation to theoretical models, to provide a more comprehensive analysis than previously presented.

Page 19-23, Line 468-567

*DISCUSSION*

*This study highlights the complexity and variety of challenges of AI implementation in clinical practice and identified six themes on which hospitals should focus when enhance the potential value of AI for patients in clinical practice. This study highlights the complexity and diversity of challenges associated with AI implementation in clinical practice, identifying six themes that span value, application, technology, ethics, and responsibility. These themes provide a comprehensive framework for hospitals to enhance the potential value of AI for patients.*

*Key findings were the importance of aligning AI development with clinical needs rather than merely pushing new technologies. This alignment is crucial for successful clinical implementation. Therefore, it is essential to move beyond generating knowledge to creating clear pathways for implementing AI models. Timely involvement and effective communication with all stakeholders are also vital for successful AI implementation. In support, hospitals play a pivotal role in this process by providing a clear vision and strategy for AI development. This includes ensuring that the necessary infrastructure and expertise are in place. Additionally, staying aligned with evolving laws and regulations is important to ensure compliance and foster innovation. By focusing on these interconnected themes, hospitals can better navigate the complexities of AI implementation and enhance its value in clinical practice.*

*Comparison with prior work*

*To our knowledge, this is the first study comprehensively explore the experiences of healthcare professionals, researchers, and Policy and Governance Experts regarding the practical implementation of AI in hospital settings. While previous studies have examined various challenges of integrating AI into healthcare, our research provides more in depth insights into the experiences by early adopters of AI technologies with the IFVAIH framework as guideline for the interviews.*

*Our findings have similarities and differences with previous studies that have explored the challenges of implementing AI in hospitals. One of the common barriers we identified is the complexity of successfully integrating AI into the workflow of healthcare providers. This is also acknowledged in a previous study, which highlights these challenges in applying AI to clinical workflows across Western and Middel Eastern countries.(24-26) However, a notable contrast between our findings and that study is the lack of fear of losing professional autonomy among the healthcare providers we interviewed in studies included in this review.(27) This may be due to the fact that our study focused on participants who are actively involved with AI implementation, resulting in more insights in the performance expectancy of AI implementation and less concerned about losing autonomy, but seeing it as an opportunity instead.*

*Furthermore, our findings are consistent with the challenges related to data quality and ethical policy issues, such as privacy, as discussed in another study, underscoring the universality of these issues when implementing AI in hospital settings.(28) For instance, data quality issues often arise from inconsistent data entry practices, lack of standardized protocols, and the integration of data from multiple sources (e.g. electronic health records and MRI scans in a separate viewer), which can lead to inaccuracies and hinder effective AI implementation. Additionally, related to ethical concerns and patient privacy are paramount, especially in regard to data accessibility for AI training. Nevertheless, these challenges are not unique to our study but are present in various implementation efforts of technological innovations, highlighting the need for robust data governance frameworks and ethical guidelines to ensure the responsible use of AI in healthcare.*

*Furthermore, the importance of policy and regulations and experience ethical and moral dilemmas with it were in line with previous studies from Europa, USA and Asia.(29,30) Although its importance was recognised, we found that awareness among health professionals and scientists was limited, as was the involvement of relevant stakeholders. This limited awareness could be attributed to the rapid pace of advancements outstripping the development and dissemination of relevant laws and regulations and training programs. In addition, the complexity and specificity of regulations may deter thorough understanding and engagement, especially among healthcare professionals primarily focused on patient care.*

*Moreover, our results were in line with another Dutch study of Kim et al.(31), which highlights that healthcare professionals broader than radiology need transparency regarding the algorithms used in AI systems. This points to the essential role of understanding and explainability of the algorithms in building trust and acceptance of AI technologies among healthcare professionals which is also important in providing information to their patients.(32)*

*Furthermore, our findings about the need for adequate interprofessional collaboration was in line with a previous review which also found teamwork as a relevant challenge for successful implementation of AI requiring strong communication, shared decision-making, coordinated actions, and progress evaluation eventually ensuring that different perspectives and expertise are integrated.(33,34). Also, emphasis on social influence and facilitating conditions further supports the need for strong interprofessional collaboration and organizational support.(32) However, achieving this level of collaboration can be challenging due to differences in professional languages, priorities, and workflows. Therefore, fostering a culture that promotes open communication, mutual respect, and continuous learning is essential for overcoming these barriers and ensuring the successful implementation of AI in healthcare settings. Here, a clear vision and strategy from the hospital may be required.*

*This role of the hospital to define a clear vision and strategy on AI development was also found in a previous study from Pakistan. ^(25)^ While this is supported by an earlier study(25), our findings highlight the need for hospitals to proactively establish the necessary infrastructure and expertise to support AI initiatives. By articulating a strategic approach to AI, hospitals can better align their resources and objectives with the technological advancements.*

10. Line 461: The fact that the participants are “pioneers of AI implementation” should be mentioned in the methods section as well as in the description of the study participants.

Revised: We acknowledge that "pioneers" may not fully capture the diversity of our participants' experiences. Instead, we changed this by describing them as individuals actively involved in AI innovation within their work. This terminology better reflects their engagement with AI technologies and their practical experience in this field. We have revised this section of the discussion.

Page 21, Line 522-526

*This may be due to the fact that our study focused on participants who are actively involved with AI implementation, resulting in more insights in the performance expectancy of AI implementation and less concerned about losing autonomy, but seeing it as an opportunity instead.*

11. Line 466: Please explain in detail what is meant by “the importance of policy and regulations and experience ethical and moral dilemmas”.

We have provided an explanation to this section.

Page 22, Line 537-544

*Furthermore, the importance of policy and regulations and experience ethical and moral dilemmas with it were in line with previous studies.(29,30) Although its importance was recognised, we found that awareness among health professionals and scientists was limited, as was the involvement of relevant stakeholders. This limited awareness could be attributed to the rapid pace of advancements outstripping the development and dissemination of relevant laws and regulations and training programs. In addition, the complexity and specificity of regulations may deter thorough understanding and engagement, especially among healthcare professionals primarily focused on patient care.*

12. Implication: I really appreciate that you want to give specific hints on how to implement AI. At the moment I am not able to connect this section with the results presented. The same problem exists with the IFVAIH model, here too I miss the connection to your results.

Thank you for this valuable comment. By incorporating the subcategories and mapping them onto the IFVAIH funnel (Table 2), we aim to clarify the connection to the implications section (Page 24). Additionally, we have expanded the implications section to explicitly link it to the domains of the funnel.

**Reviewer #2:** Overall, this is a topical and interesting study on the challenges of implementing AI in clinical practice. However, the manuscript would benefit from further proofreading. Some errors have been highlighted below, but this is not exhaustive.

Introduction

13. CE- marked? Please spell out acronym

We have clarified the acronym.

Page 5, Line 105-107

*More than half of CE-marked (Conformité Européenne) medical AI devices between 2015 and 2020, certified for compliance with EU safety and performance standards, were intended for use in imaging.*

14. How do these five domains (Value, Application, ethics technology and responsibility ) map onto figure 1?

Each phase of the funnel in Figure 1 addresses the five domains (Value, Application, Ethics, Technology, and Responsibility). This integrated approach ensures that all relevant aspects are considered at every stage of AI implementation, providing a comprehensive framework for evaluating and enhancing AI readiness in hospital settings. We have clarified this in the text.

Page 5, Line 122-134

*To address this gap between promise of AI and its clinical implementation, a framework has been proposed to guide AI initiatives from ideation to clinical implementation. This framework, the Innovation Funnel for Valuable AI in Healthcare(21) (IFVAIH) (Dutch: Waardevolle AI tool) (Figure 1), offers a comprehensive checklist for researchers and developers in advancing valuable applications from inception to scalability, this tool provides guidance to the legal and regulatory parameters governing their actions. This allows an early start to prepare for minimum requirements or standards and to reflect on actions to proceed in the IFVAIH. In the seven-phase funnel, which includes the five pervasive domains of Value (ensuring AI initiatives provide clear benefits) , Application (practical use of AI in clinical settings), Ethics (ethical considerations and adherence to ethical standards), Technology (leveraging technological capabilities), and Responsibility (accountability and responsibility of AI use), each phase has a clear goal and encourages creative work while using resources wisely, providing a structured approach that helps strategize to meet standards and develop human-centric AI applications for clinical practice.*

15. Is figure 1 colour coded? (I.e does the red vs blue vs yellow have meaning)?

The colours used in the figure are used by the official funnel but have no meaning.

16. Also, figure is repeatedly described as a “funnel” which suggests a pyramid structure, but then the figure is linear and implies the process is uni-directional.

Revision: Thank you for your insightful comment. The term "funnel" is the original name of the model, and we acknowledge that it may suggest a pyramid structure. We agree with your observation and have updated the figure that more clearly illustrates the linear and uni-directional nature of the process. However, we prefer to retain the original name "funnel" as it reflects the comprehensive journey that many innovations undergo through the entire process. This terminology also emphasizes the structured approach and the narrowing focus as innovations progress through each phase.

Method

17. It would be useful to get more information on the participant selection and sampling procedure, answering the following questions:
    - Participants were contacted by email, but how were these emails obtained?

We used purposeful sampling through the network of the researchers. We have clarified this in the manuscript.

- Was the study advertised somewhere?

We did not advertised the study. We have clarified this in the manuscript. Together with the previous comment, we hopefully become more clear how participant selection and sampling was conducted.

- Where participants known to the research group?

Yes, some participants were known to the research group but these were no direct colleagues (within the hospital, which has a large workforce of approximately 7,000 employees). This familiarity can have advantages, such as facilitating trust and openness during interviews. We have clarified this in the manuscript.

- Was there a screening survey used to identify eligible participants? Where their potential participants who were interested but not eligible?

The screening process involved purposefully asking individuals within our network who met the eligibility criteria. This targeted approach allowed us to efficiently identify suitable participants. Although we did not use a formal screening survey, as the only eligibility criterion was having prior experience with AI initiatives, this method ensured that we included participants who were well-suited for the study.

Page 7, Line 164-170

*Participant selection*

*Participants were purposeful sampled through the network of the researchers and contacted by email; a reminders were was sent if there was no response within two weeks. The study was not advertised by regular communication channels of the hospital. Although participants worked in the same hospital (of approximately 7000 employees), there were no direct or prior colleagues or relationships with the participants. A minimum of four participants per subgroup was deemed sufficient to explore the major experiences and reach data saturation. We scheduled the interviews after informed consent.*

18. A flow diagram might be useful.

We chose not to include a flow diagram due to the low complexity of our sampling process, which involved directly contacting individuals within our network who met the single eligibility criterion of having prior experience with AI initiatives. This straightforward approach made a detailed flow diagram unnecessary, and we believe our extended description sufficiently clarifies the steps taken to identify and select suitable participants.

19. In line with COREQ guidelines, could a small section on reflexivity be added- how did the interview moderators’ backgrounds influence the interview dynamic and analysis?

In line with COREQ guidelines, we have added a section on reflexivity to our analysis. Our interview team comprised a combination of clinical and technical experts, including a nurse scientist and a technical PDeng. This diverse team conducted the interviews, coded the data, and subsequently developed themes from a broad range of perspectives.

We believe that the backgrounds of the interview moderators influenced the interview dynamics and analysis in several ways. The clinical background of some moderators likely facilitated rapport and recognition among participants, fostering open discussions about clinical challenges and opportunities. Similarly, the technical PDeng's background would have encouraged in-depth conversations on technology and data-related topics. This complementary expertise provided a holistic view of the issues at hand.

However, we acknowledge that the interviews may have been unconsciously steered towards topics more familiar to the interviewers, potentially introducing confirmation bias. To mitigate this, we cross-checked our findings with other team members to ensure a balanced and comprehensive analysis.

Page 8, Line 190-210

*To analyse the interviews, we used a six-step thematic analysis^(23)^ using the qualitative data analysis software ATLAS.ti 23.0 (Cleverbridge AG, Cologne, Germany). We used this approach to maintain openness and flexibility in identifying emerging themes based on the codes during the analysis of the data collected. The stages included: (1) immersion; (2) generating initial codes; (3) searching for and identifying themes; (4) reviewing themes; (5) defining and naming themes; and (6) writing the report. Stage 1 to 3 were conducted independently by two researchers (JL and MtH). During the first and second stage, JL (clinical background) and MtH (technical background) became familiar with the data by listening to the audiovisual recordings, checking the transcriptions against the audio recording, reading, listening sections again and re-reading the final transcripts. During the third stage, both researchers read the transcripts and codes for categorizing similar statements into first themes. For the fourth and fifth stages, JL, MtH, PH (technical background), AJ (clinical background), GH (technical background) were responsible for reviewing, defining and naming themes, which were discussed with all authors. Also the formed themes were mapped onto the five domains (value, application, ethics, technology, and responsibility) of the IFVAIH funnel. The diverse background of the interview moderators and researchers ensured a comprehensive and holistic perspective on AI implementation. The clinical background of the researchers likely facilitated rapport and recognition among participants, thereby fostering open discussions about clinical challenges and opportunities. Conversely, the technical background likely encouraged in-depth conversations on technology and data-related topics. During the sixth stage, the themes were brought to participants for member checking by e-mail, which did not result in any changes to the themes.*

Results

20. Age and gender play a large role in technology acceptance. It might be useful to add these columns to table. To minimise deductive disclosure, age categories rather than raw ages could be used. It would also be useful to reflect on how these factors influence the likelihood of being “early adopters” of AI.

We do agree such factors may be of influence on the adoption of novel technologies such as AI. Although the sample size is small to draw conclusions on these factors in relation to our data, we have added the sex of the participants to Table 1. Unfortunately, we did not collect the age of the participants. We extended the discussion section related to age and sex.

Page 23, Line 570-580

*Strengths of the study include the depth, diversity, and quality of the data, which collectively provide a comprehensive and in depth view of current practice and allows for a nuanced understanding of the interplay between social and technical factors, highlighting the importance of considering both elements.(35) However, a few limitations that may influence the interpretation of the results. First, the context of this study was confined to one specific hospital. While this hospital is actively developing AI models and thus offers valuable insights, other hospitals with different infrastructures, cultures, and policy frameworks might encounter different challenges and experiences with AI implementation. Additionally, it may be of interest to include factors such as age and sex in a broader (quantitative) evaluation as these factors may be of influence in technology acceptance.(32) Nevertheless, it is important to note that generalizability is often not a primary aim of qualitative studies.(36)*

21. The sample has a wide range of professional diversity, which is great to see.

Thank you for this feedback.

22. Could an additional table showing the codes developed and which themes they informed be added? This would add greater transparency to the thematic analysis process.

We have chosen not to present the codes in the main manuscript because, although it may offer more transparency, it may also lead to information overload due to the iterative and continuous nature of our coding process, which involved multiple rounds of merging and revising. This process does not result in a single, definitive set of codes. Instead, we have added table in the manuscript (Page, Table 2) included sub-themes that provide clarity and enhance understanding without overwhelming the reader.

Page 23, Line 285

**Table 2: Overview of themes, subcategories and mapping onto the IFVAIH Funnel**

| **Theme** | **Subcategories** | **Domains of the IFVAIH** |
| --- | --- | --- |
| Balancing between demand-pull and tech-push | Tech-push by examples and commercial parties | Value |
|  | Experience with AI needed for demand-pull | Value |
|  | Demand-pull factors | Value |
| Focus on generating knowledge, not implementing working solutions | Unclear goal definition and required steps for implementation | Value, Application |
|  | Focus on research vs. focus on clinical practice | Value, Application |
| Lacking multidisciplinary collaboration | Stakeholders are not or only involved in later stages | Application, Technology, Ethics, Responsibility |
|  | Variety of perspectives on AI development and implementation | Application, Technology, Ethics, Responsibility |
| Lack of appropriate knowledge and skills | Of requirements of AI development | Technology |
|  | Of ethical, legal and societal aspects of AI development | Ethics, Responsibility |
|  | Of added value in clinical practice of AI development | Value, Application |
| The role of the hospital in AI-development | Vision and strategic planning | Value, Reponsibility |
|  | Technical requirements and necessary infrastructure | Technology |
|  | Responsibility as AI manufacturer | Responsibility |
| Opportunities and challenges from evolving laws and regulations | Increasing safety and reliability | Responsibility, Ethics |
|  | Lack of clarity and unfamiliarity with new regulations | Responsibility |
|  | Integration in clinical guidelines | Application, Responsibility |

**Abbreviations: IFVAIH: Innovation Funnel for Valuable AI in Healthcare**

23. When giving participant quotes, could the participant professions be added alongside their ID, for increased readability and clarity?

Revised: We have added this to every quote throughout the manuscript.

24. Line 253: should say “plan on how to”

We have revised this sentence accordingly.

25. Line 379: should be a capital A, at the start of the sentence

We have revised this sentence accordingly.

Discussion

26. Lines 515 to 518 are a repeat of lines 512 to 514.

We have deleted this repetition.

27. Under “comparisons with prior work” it would be useful if the geographical and temporal context was given to the studies cited, as developments and opinions of AI are happening at a rapid pace.

Thank you for this valid comment. We have added these contextual factors of the primary studies and reviews under this section when reported.

Page 21-23

**Reviewer #3**

28. The authors identify six themes that are clearly defined and supported with the results. How do these challenges connect with the Innovation funnel presented in the introduction? It is valuable to bring the results into the framework. Perhaps linking each theme/challenge to a stage for action in the funnel.

By adding the subthemes and mapping them onto the domains of the funnel in Table 2, we tried to bring the results into the framework. Given the domains are relevant for each stage of the funnel, we did not map this to specific stages.

Page 23, Line 285, Table 2

29. **What are the strengths of the study? Future steps?**

We have added the strength of study and formulated next steps besides the implications section (Page 24, Line 599-658).

Page 23, Line 570-180

*Limitations*

*Strengths of the study include the depth, diversity, and quality of the data, which collectively provide a comprehensive and in depth view of current practice and allows for a nuanced understanding of the interplay between social and technical factors, highlighting the importance of considering both elements.(35) However, a few limitations that may influence the interpretation of the results. First, the context of this study was confined to one specific hospital. While this hospital is actively developing AI models and thus offers valuable insights, other hospitals with different infrastructures, cultures, and policy frameworks might encounter different challenges and experiences with AI implementation. Additionally, it may be of interest to include factors such as age and sex in a broader (quantitative) evaluation as these factors may be of influence in technology acceptance.(32) Nevertheless, it is important to note that generalizability is often not a primary aim of qualitative studies.(36)*

30. **Authors could review a couple of publications about the sociotechnical contexts. The context is mentioned as a limitation, how could this be explained/strengthened from a sociotechnical perspective.**

We have reviewed relevant publications on sociotechnical contexts and incorporated this perspective throughout our discussion (limitations, comparison with prior work, implications). We have expanded on how the sociotechnical context influences AI implementation. This includes discussing the interplay between social factors, such as stakeholder collaboration and organizational culture, and technical factors, such as IT infrastructure and data management.

Page 23, Line 570-572

*Limitations*

*Strengths of the study include the depth, diversity, and quality of the data, which collectively provide a comprehensive and in depth view of current practice and allows for a nuanced understanding of the interplay between social and technical factors, highlighting the importance of considering both elements.(35)*

Page 25, Line 649-658

*Investing in mutual understanding between stakeholders*

*Drawing on theme 3, an important aspect of successful AI implementation in hospitals it's crucial to foster long-term collaboration among physicians, technologists, researchers, and Policy and Governance Experts. Building enduring relationships is more beneficial than seeking short-term partnerships. To achieve this, integrate social learning into the SOP, such as using responsive evaluation to gather stakeholder perspectives, facilitate mutual learning, and assess AI impact.(42) Methods like action research or design thinking can help map changes and experiences. This includes discussing the interplay between social factors, such as stakeholder collaboration and organizational culture, and technical factors, such as IT infrastructure and data management.(35) Ultimately, establishing trust and expertise through these collaborations is key to successful implementation of AI initiatives.*

31. **Overall all sections are very well presented.**

Thank you for your feedback.

32. **No suggestions of changes for the methodology. It is very well structured and corresponds to a high standard of qualitative research.**

Thank you for your feedback.
